# “But Thanks to God, I Learned to Forgive Them and Work on Myself and Things That I Love”: Revenge Fantasies of Indian Muslim Women through Drawings and Narratives

**DOI:** 10.1007/s11013-026-10009-0

**Published:** 2026-07-23

**Authors:** Meghna Girish

**Affiliations:** Graduate School of Creative Arts Therapies, University of Haifa, Haifa, Israel

**Keywords:** Revenge fantasies, Women, Indian, Drawings, Interpretative phenomenological analysis

## Abstract

This study explores how women make sense of injustice and express revenge fantasies through both visual and narrative forms of expression. Using an interpretative phenomenological approach, the study examines drawings and accompanying narratives from fourteen Muslim women in Kerala, India, an under-researched population within the literature on revenge and coping. The analysis identified four superordinate themes: emotional validation through relational reversal, restoration of moral order through indirect or inevitable justice, reclaiming power through aggression and achievement, and withdrawal, moral reflection, and transformation of response. Findings suggest that revenge fantasies are not uniform or solely driven by aggression. Rather, they reflect a range of psychological processes aimed at restoring balance, agency, and meaning following unjust experiences. Across themes, participants were often not positioned as direct agents of revenge but instead engaged in imagined, symbolic, or internally oriented responses. The integration of drawings and narratives revealed important distinctions in expression: Drawings captured more immediate and affective dimensions, while narratives reflected more structured and socially mediated interpretations. Convergences between modalities indicated clarity in meaning-making, whereas divergences highlighted internal conflict and evolving interpretations. The study contributes to the existing literature by demonstrating the value of combining visual and verbal methods for understanding complex emotional experiences. It also offers preliminary insights into how socio-cultural and religious contexts may shape responses to injustice. Overall, revenge fantasies emerge as dynamic and multifaceted processes that function less as intentions to harm and more as ways of negotiating emotional pain, identity, and psychological balance.

## Introduction

Experiences of injustice are a pervasive aspect of human life, evoking complex emotional responses such as anger, hurt, and moral outrage (DiBlasi & Kassinove, 2022; Elshout et al., 2017). Among these responses, revenge fantasies occupy a psychologically significant yet understudied space. Rather than being understood solely as indicators of aggression or maladjustment, revenge fantasies can be viewed as internal processes through which individuals make sense of harm, restore a sense of agency, and symbolically negotiate violations of fairness (Girish & Lev-Wiesel, 2024b, 2025; Goldner et al., 2019; Recchia et al., 2019; Zukerman et al., 2023). They represent not only emotional reactions but also forms of meaning-making that allow individuals to engage imaginatively with experiences that might otherwise feel overwhelming or unresolved. However, the ways in which such fantasies are experienced, constructed, and expressed are not culturally neutral. Revenge fantasies can thus be understood as culturally regulated, morally negotiated, and symbolically expressed responses to experiences of injustice.

A growing body of work within cultural psychiatry and social psychology has emphasized that emotional expression is deeply embedded within socio-cultural contexts (Cohen et al., 1996; Osgood, 2017; Yoshimura & Boon, 2018, 2023). Cultural norms shape what emotions are considered acceptable, how they are expressed, and the meanings attributed to them. Experiences of anger, justice, and retribution, in particular, are often regulated by culturally specific moral frameworks informed by social, religious, and historical contexts (Gelfand et al., 2012). As such, revenge fantasies cannot be fully understood without attending to the cultural systems within which they emerge (Shteynberg et al., 2009). Within these cultural systems, gender and religion function as powerful organizing forces that shape individuals’ responses to injustice. Gendered socialization often influences how emotions such as anger and aggression are experienced and expressed, with women frequently navigating expectations of restraint, relational sensitivity, and moral accountability, while men are likely to engage in retaliatory forms of violence (Goldner et al., 2019; Yoshimura & Boon, 2023). Religious frameworks, in turn, provide moral scripts that may simultaneously discourage retaliation while offering alternative interpretations of suffering, justice, and forgiveness (Sandage et al., 2003; Schultz et al., 2010). In India, Muslim women occupy a particularly complex socio-cultural position, situated at the intersection of gendered expectations and minority status. Existing literature has paid limited attention to how revenge fantasies are shaped by culturally embedded moral frameworks, particularly within contexts where expressions of anger are both socially and religiously regulated.

Muslims represent the largest minority community in India, within which Muslim women occupy a particularly complex socio-cultural position shaped by intersecting structures of gender, religion, and marginalization (Das, 2025; Narain, 2023). Existing research suggests that Muslim women experience persistent disadvantages across educational, economic, and social domains when compared with several other social groups in India (Ohlan, 2020). For instance, only 13 out of 100 Muslim women enroll in higher education (Sulthan, 2025), with Muslim women being described as among the least empowered groups with respect to education, employment, and social participation (Sanu, 2018). Workforce participation among Muslim women remains particularly low, with Muslim women accounting for only 10% of the female urban workforce in India (Malik, 2023). These disparities are not solely attributable to religion but emerge through the intersection of gender, minority status, socio-economic disadvantage, geographic location, and intra-community stratification (Bazaz & Akram, 2021; Rahiman, 2023). These inequalities also extend to family and marital decision-making. For example, over 40% of forcefully conducted marriages among Muslim women were attributed to family preference for early marriage, a practice associated with reduced educational attainment and diminished livelihood opportunities later in life (Rajasenan et al., 2022). Such processes of minoritization shape access to opportunities, experiences of inclusion and exclusion, and the broader social contexts within which injustices are experienced and interpreted.

Prominent representations of the “Muslim woman” as passive and oppressed obscure the diversity and complexity of Muslim women’s lived realities across contexts. Their experiences are shaped by intersecting structures of gender, religion, and minority status, often within socio-political environments that are hostile to their well-being, while simultaneously reflecting forms of agency and negotiation in everyday life (Jeffery & Qureshi, 2022). Muslim women in India often navigate a dual reality marked by both emerging avenues for empowerment and persistent structural barriers, including economic marginalization, cultural constraints, and experiences of harassment (Bhimdiwala et al., 2024). Bhimdiwala et al. (2024)’s study explored how Muslim women experience gendered and faith-based harassment as cumulative and systemic, while engaging in collective processes of sensemaking to interpret and respond to such harm (Bhimdiwala et al., 2024). Women's responses were shaped by social and spiritual resources, including religious practices that support resilience and a moral orientation in the face of injustice. An important aspect lies in the early socialization of girls into religious teachings, often beginning in childhood through Madrasa education and reinforced within broader socio-cultural expectations such as early marriage (Das, 2025). As these frameworks are internalized over time, they may meaningfully shape how injustice is perceived and negotiated, including how responses such as revenge or retribution are imagined and morally evaluated.

Responses to injustice can vary considerably, ranging from forgiveness, avoidance, and reconciliation to retaliation and desires for revenge (DiBlasi & Kassinove, 2022; Goldner et al., 2019). Such responses are often shaped by how individuals interpret the harm experienced, the power imbalance created by the event, and the perceived possibility of restoring justice (Elshout et al., 2017; Goldner et al., 2019). Revenge and vengeful thoughts may, therefore, serve as attempts to symbolically re-establish power, agency, or moral balance following experiences of violation or unfair treatment (Haen & Weber, 2009; Lillie & Strelan, 2016; Seebauer et al., 2014). In this sense, revenge extends beyond a purely emotional reaction and becomes a moral judgment concerning what constitutes a fair or appropriate response to wrongdoing (DiBlasi & Kassinove, 2022; Gelfand et al., 2012). Religious traditions provide frameworks through which concepts such as justice, punishment, forgiveness, and retaliation are understood and evaluated (Sandage et al., 2003; Schultz et al., 2010). Religion or religious affiliation may provide an important contextual lens for understanding revenge fantasies, as religious teachings and moral traditions often shape broader beliefs about justice, forgiveness, retaliation, and reconciliation. However, the extent to which these frameworks are reflected in individuals' experiences remains an empirical question.

Within Islamic moral frameworks, responses to harm are not understood purely in terms of emotional reaction but are situated within a broader ethical system that emphasizes justice, restraint, and moral accountability. While Islamic jurisprudence permits retaliation in the form of *Qisas* (proportionate retribution), and the perpetrator experiences the same suffering as the victim (Asif Khan, 2022), it simultaneously privileges forgiveness, patience (*sabr*) (Arif & Sarwar, 2025; Salama, 2024), and the regulation of anger as a higher moral ideal (Karim et al., 2017). These principles position individuals within a tension between the desire for retribution and the expectation of moral restraint, where enduring harm without retaliation is often valorized. Consequently, experiences of injustice may not only evoke desires for revenge but also require individuals to negotiate these desires within internalized religious and ethical frameworks. This reinforces the view that revenge fantasies are culturally situated and morally negotiated responses to injustice. It may also create a psychological and moral tension, wherein individuals must navigate the pull between emotional responses to injustice and internalized expectations of restraint and forgiveness.

Revenge fantasies operate as cognitions that emerge after being mistreated and can have a coping and regulatory function (Haen & Weber, 2009; Horowitz, 2007). They can offer a momentary sense of empowerment or control, addressing the feelings of frustration, humiliation, and insult by virtually punishing the perpetrator and settling the score. These fantasies could have a pseudo-power effect, which can be cathartic (Lillie & Strelan, 2016; Seebauer et al., 2014). In this sense, revenge fantasies may also serve as symbolic acts, allowing individuals to reclaim a sense of agency in contexts where direct retaliation is constrained. These imagined acts of retribution may thus serve as psychologically safe spaces for the articulation of otherwise constrained emotions. In communities and societies where individuals feel like the minority and are unable to externalize their authentic feelings, such fantasies may also operate as an internal and private space to release and process suppressed emotions. This could be particularly relevant for Muslim women in India, as the literature repeatedly recounts how women engage in patterns such as self-silencing to maintain relationships and avoid confrontation during conflicts (Akram et al., 2024; Naeem & Ehsan, 2025; Sharma et al., 2025). Having to navigate through restrictive or patriarchal environments, where overt expressions of rebellion or anger may be met with punishment, what role could revenge fantasies play among this people group?

Despite the relevance of these dynamics, existing research on revenge and responses to injustice has been largely limited by its reliance on Western samples and predominantly verbal, questionnaire-based methodologies (McGaughey et al., 2025). Such approaches may not adequately capture culturally embedded or non-verbal expressions of distress, particularly among populations in which direct articulation of anger or retaliation may be constrained. This creates a critical gap in the literature, where the inner worlds of individuals—especially those from marginalized backgrounds—remain only partially understood. Art-based methods offer a valuable alternative by providing access to symbolic, non-verbal, and often less consciously mediated forms of expression (Goldner et al., 2021; Karp, 1997; Malchiodi, 2020). Drawings, in particular, can function as a projective medium through which individuals externalize internal experiences, allowing the representation of emotions, conflicts, and imagined scenarios in ways that may not be easily verbalized (Girish & Lev-Wiesel, 2024a; Lev-Wiesel & Liraz, 2007; Lev-Wiesel et al., 2023). When combined with narrative accounts, these visual expressions can be contextualized and elaborated, enabling a more comprehensive understanding of meaning-making processes (Bhattacharyya et al., 2019; Lev-Wiesel & Liraz, 2007). Together, drawings and narratives offer a complementary, multi-layered approach that extends beyond traditional methodologies and allows for the exploration of culturally situated expressions of distress and response.

The present study builds on this methodological and theoretical framework by examining revenge fantasies among Muslim women in India through an integrated analysis of drawings and narratives. Positioned within a culturally informed, qualitative approach, the study investigates how these women represent and reimagine experiences of injustice across visual and verbal modalities. By focusing on an underrepresented population and employing a non-intrusive, art-based methodology, this research seeks to illuminate how socio-cultural, gendered, and religious contexts shape both the experience of harm and the imagination of response. Consistent with an interpretative phenomenological approach, the study seeks not only to describe these experiences but to interpret how participants make sense of them within their socio-cultural and moral worlds. While previous work (Author, 2025) examined revenge fantasies and injustices using a mixed-methods approach in a broader sample comprising participants from multiple religious backgrounds, the present study focuses exclusively on Muslim women and is based on an independent dataset collected during the same broader research period. These data were not included in the earlier publication and were analyzed separately to address a distinct research question concerning the symbolic representation, meaning-making, and psychological functions of revenge fantasies. Consequently, the present study extends this line of work by providing a more focused and culturally situated understanding of how experiences of injustice and imagined responses to them are represented and negotiated among Muslim women.

Based on the above aims and review, the research questions for the study were: (1) How do Indian Muslim women experience and make sense of injustice through drawings and narratives within their socio-cultural and religious contexts? (2) How are revenge fantasies expressed, and how are they interpreted and negotiated as part of these women’s lived experiences? and (3) How do drawings and narratives converge or diverge in representing and making sense of injustice and revenge?

## Materials and Methods

The present study employed data analysis guided by an interpretative phenomenological framework. This framework draws on Interpretative Phenomenological Analysis (Nizza et al., 2021; Smith, 2017), grounded in a hermeneutic-phenomenological epistemology. Human experience is looked through as inherently interpretative, relational, and enclosed within individuals’ ongoing engagement with their lived world (Smith, 2019). There are layers of lived experience that may not be fully captured through verbal expression alone, but can be accessed via artworks (Boden et al., 2019; Day et al., 2024).

The present study’s analytic process attends to both participants’ subjective meaning-making and the researcher’s interpretative role in co-constructing meaning (Barrett-Rodger et al., 2023; Smith, 2019). The drawings were treated as autonomous expressive texts, carrying distinct symbolic and affective qualities. These visual materials were understood as communicating emotions, experiences, and implicit meanings through elements such as form, texture, and imagery (Boden et al., 2019; Huss et al., 2012). At the same time, visual and verbal data were examined together rather than separately, following an integrative approach, aligned with a hybrid repertoires framework. Specific attention was given to how meaning emerged through the dynamic interplay between drawings and accompanying narratives (McCullough & Lester, 2023). Such an approach was particularly suited to the present study, which aimed to explore how individuals make sense of injustice and articulate revenge fantasies through both visual and narrative forms of expression.

## Participants and Procedure

A total of 14 women aged between 19 and 27 years (*M* = 20.64; *SD* = 2.56), who identified as Muslim and hailed from Kerala, participated in the study. The data for the present study were collected during the same broader research period as that described in Girish & Lev-Wiesel (2025), but represent a distinct sample not included in the prior analysis. Data were collected through a private online survey platform, allowing participants to complete the drawing and narrative tasks independently and reducing potential influences from the researcher or other participants. Inclusion criteria required only that participants be Muslim women with Indian citizenship residing in Kerala. Participant recruitment followed convenience and snowball sampling. The call to participate in the research study was posted on several social media platforms and social networks. Only 14 participants showed interest, complied with the research protocol, and completed all measures. With regard to marital status, all participants were single. Only two participants (14.28%) out of the fourteen were Master’s students; the remaining participants were undergraduate college students. Trauma Events Checklist showed that verbal or emotional abuse (57.14%) and loss of a family member (50%) were the most commonly listed traumatic events. The study received ethics approval from the University of Haifa, and informed consent was obtained. Participants were contacted via online means, i.e., they were sent a Google form link with the option to upload their drawings and narratives.

## Measures

### Drawings and Narratives

Participants were asked to do a two-part task intended to explore their experiences of injustice and associated revenge fantasies. In the first part, participants were asked to create a drawing depicting an unjust event they had personally experienced. In the second part, they were asked to create a separate drawing showing what they would like to happen to the perpetrator of the injustice, thereby representing their revenge fantasy. Following each drawing, participants were asked to provide a written narrative describing the content and meaning of their image. The task was kept open-ended to allow for individual interpretation and expression, enabling participants to communicate their experiences through both visual and verbal modalities. This multimodal approach facilitated the exploration of both explicit and implicit aspects of meaning-making, capturing emotional, symbolic, and relational dimensions that may not be fully accessible through verbal accounts alone (Boden et al., 2019).

### Trauma Events Checklist

A checklist of nine specific types of traumatic events from the Traumatic Events Questionnaire (Vrana & Lauterbach, 1994), namely, car accident, physical abuse, terror attack or war, hospitalization due to illness, sexual abuse, loss of family member, social exclusion, verbal or emotional abuse, and other events. Participants could mark more than one event, and each event was coded as one if it occurred and zero if it never happened. The number of events reported was summed to determine the severity of the trauma history. The checklist provided background information on participants’ exposure to adverse life events, allowing for a broader understanding of the contexts in which revenge fantasies emerged.

### Demographics Sheet

Demographic data, including age, level of education, and marital status, were collected.

## Data Analysis

The data were analyzed following the principles of interpretative phenomenological analysis (Smith, 2017, 2019), involving an iterative and inductive process of engaging with both visual and narrative materials (Boden et al., 2019). Each case was examined individually to preserve its idiographic depth, beginning with repeated readings of the narratives alongside detailed observation of the corresponding drawings (McCullough & Lester, 2023). Initial noting involved descriptive, linguistic, and conceptual comments, attending to both the explicit content and the underlying meanings conveyed through visual and verbal expression. Drawings were analyzed in relation to their symbolic, spatial, and affective qualities, including the use of figures, positioning, boundaries, and other visual elements. Narratives were also read alongside notes generated. Subsequently, emergent themes were developed for each participant by integrating insights from both modalities, focusing on how individuals made sense of their experiences of injustice and articulated revenge fantasies.

Particular attention was paid to the convergence and divergence between drawings and narratives, allowing for a deeper understanding of both expressed and implicit meanings. Once case-level analyses were completed, patterns were identified across participants by clustering-related themes, resulting in superordinate themes that captured shared psychological processes while retaining sensitivity to individual variation.

A reflexive stance was maintained throughout the analytic process, with the researcher engaging in ongoing self-reflection regarding personal assumptions, emotional responses, and interpretative decisions. A reflexive journal was also maintained, noting reactions to drawings, emotional responses, possible assumptions, and decisions about coding/themes. Regular revisiting of the data, particularly the relationship between drawings and narratives, enabled a careful consideration of both convergence and divergence, ensuring that meanings were grounded in participants’ accounts rather than imposed by the researcher (Biggerstaff & Thompson, 2008; Eatough & Smith, 2017). Particular care was paid to assumptions that revenge fantasy is aggressive or retaliatory, as well as to expectations that religious themes would be prominent, given the composition of the sample. As the analysis progressed, participants' emphasis on acknowledgment, moral correction, achievement, withdrawal, and transformation challenged any assumptions, as did the relative absence of explicit religious references. Reflexive notes were used to distinguish between interpretations grounded in the data and those influenced by prior theoretical or clinical understandings. This process contributed to the refinement of themes and supported interpretations that remained closely aligned with participants' visual and narrative representations. To further strengthen the trustworthiness of the analysis, a senior researcher with expertise in qualitative research conducted periodic reviews of coding and theme development, offering critical input and ensuring that interpretations remained grounded in the data.

## Results

Analysis of both the drawings and narratives showed how participants processed personal experiences of injustice and expressed revenge fantasies against the perpetrators. Four superordinate themes yielded from the analysis were: (1) validation through reciprocal emotional experience, (2) restoring moral order through indirect or inevitable justice, (3) reclaiming power through aggression and achievement, and (4) revenge as withdrawal, moral reflection, and transformation. Table 1 summarizes the results along with participant information.

**Table 1:** Overview of Participants, Reported Injustices, and Brief Narrative Excerpts

Theme	Participant	Trauma events checklist	Excerpt from the narratives
Validation through reciprocal emotional experience	P1	Sexual abuse, loss of family member, social exclusion, verbal/emotional abuse	“The ones who passed those comments going through the exact situation and understanding the pain behind it.”
	P4	None	“She has to give the trophy to me.”
	P9	Hospitalization due to illness, loss of family member	“The teacher will allow the student to get inside the class for the same scenario.”
	P12	Loss of family member, verbal/emotional abuse	“And the lightning for a wish that I have the person knows the intensity of it”
Restoration of moral order and indirect justice	P5	Loss of family member	*“Giving and taking.”*
	P10	Verbal/emotional abuse	“Was like my brother till that day. He has to get married to a girl who is really younger than him.”
	P11	Social exclusion, verbal/emotional abuse, other	“I wanted that guy... to just be a good guy, not be with me.”
	P14	Social exclusion, verbal/emotional abuse, other	“But thanks to god, i learned to forgive them and work on myself and things that i love. And yes im happy now.”
Reclaiming control through aggression and achievement	P2	Hospitalization due to illness, sexual abuse, social exclusion, verbal/emotional abuse	“I want him to die”
	P7	Sexual abuse	“men/women causing the abuse to be treated by the society with punishment with beating”
	P13	Loss of family member, social exclusion, verbal/emotional abuse	“I should've told my mother about the incident so that I'd never have to see him again. “
	P6	Hospitalization due to illness, loss of family member, social exclusion, verbal/emotional abuse	“I really hate the person who misbehaved with me.”
Withdrawal, reflection, and transformation	P3	Hospitalization due to illness	“How I spend my painful times.”
	P8	Hospitalization due to illness, loss of family member	“It's not my personal experience but I'm a person who saw that person who was treated that way.

### Theme 1: Validation Through Reciprocal Emotional Experience

For several participants, revenge fantasies functioned as a way of validating and acknowledging their emotional pain and suffering alongside the reversal of the power imbalance that happened in the unjust event. The perpetrator was imagined to experience the same pain or exclusion that the participant originally endured. This form of revenge did not involve physical aggression but focused on emotional mirroring and role reversal. Participants who experienced relational invalidation or exclusion often expressed a desire for the perpetrator to “understand” or “feel” the same emotional impact. For instance, one participant who described peer-based invalidation of achievement imagined a scenario in which those peers would undergo a similar experience and recognize the associated pain. Similarly, participants who experienced interpersonal harm depicted revenge as a reversal of social positioning, shifting from being excluded to others experiencing the same exclusion but without them being in the picture or being the cause of their exclusion.

**Fig. 1.**
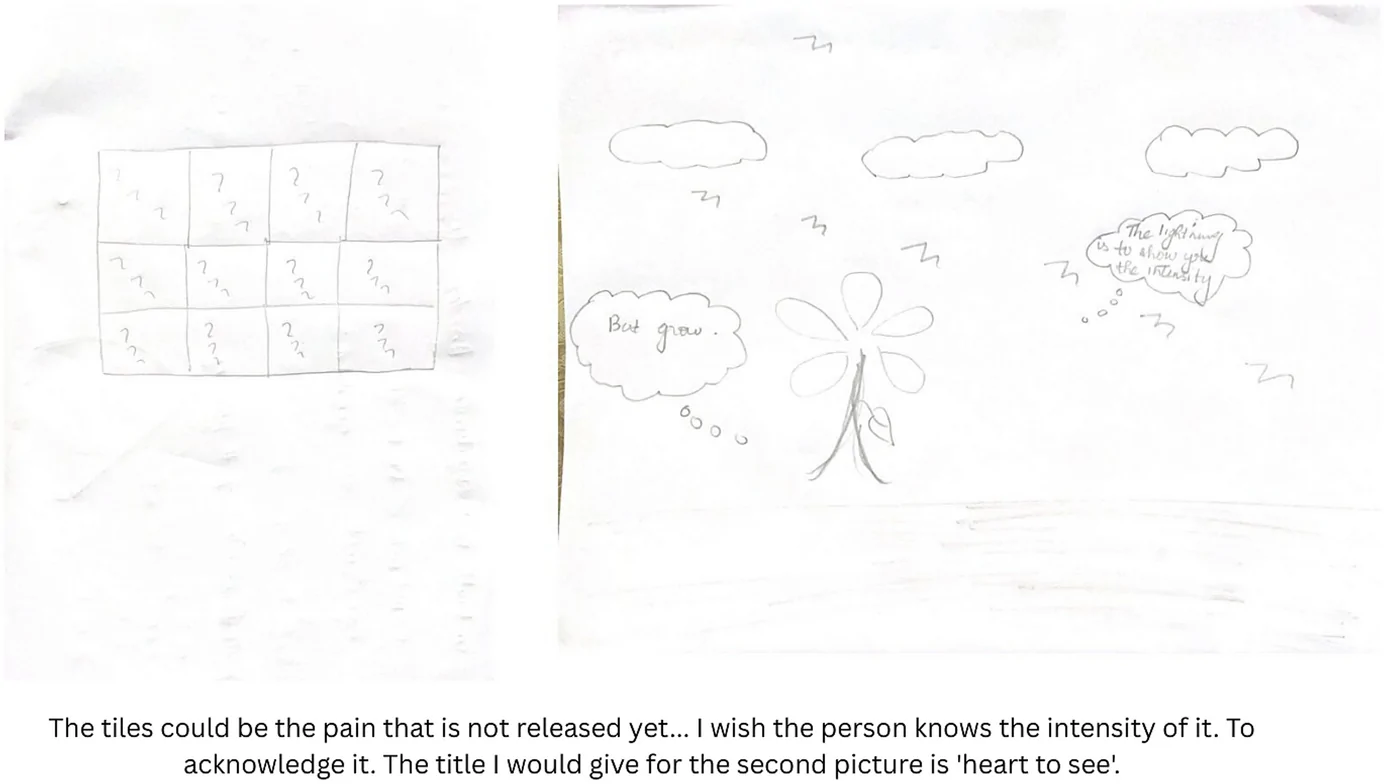
Unjust Event (left) and Revenge Fantasy (right) drawings of participant aged 24 years

**Fig. 2.**
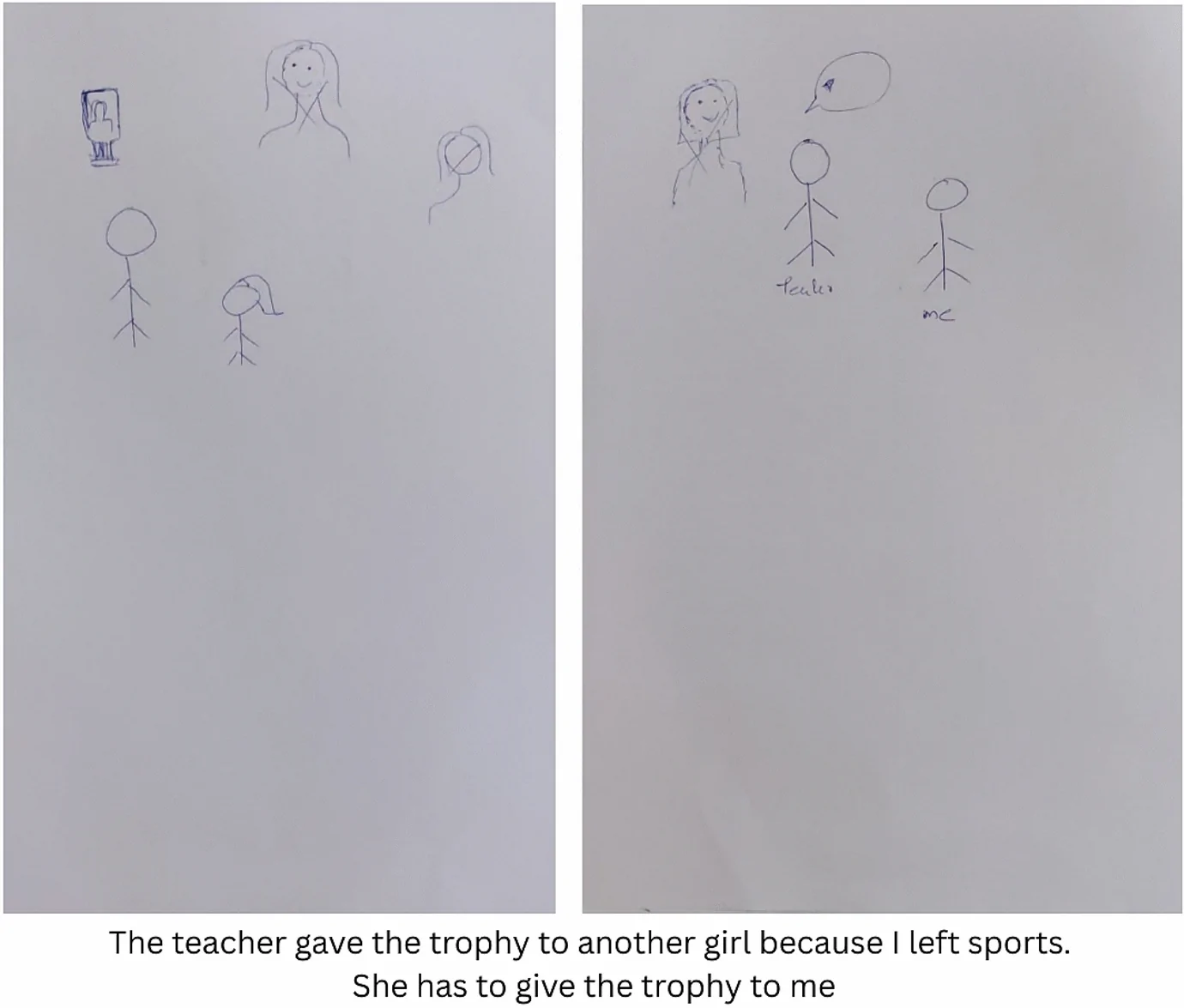
Unjust Event (left) and Revenge Fantasy (right) drawings of participant aged 20 years﻿

Drawings frequently depicted group dynamics with a focus on environmental setup, spatial separation, and shifts in positioning, such as the omission of oneself and the contrast between isolated figures and clustered groups. In revenge drawings, these dynamics were often inverted, suggesting a reconfiguration of belonging and power. The need for emotional validation and social reversal was visually depicted through the rearrangement of the environment and the restructuring of figure placement (see Figs. 1 and 2). While narratives articulated this as a desire for understanding or empathy, drawings conveyed the emotional intensity of exclusion and its reversal more implicitly. Narratives of the revenge fantasy drawing included statements like, “The ones who passed those comments going through the exact situation and understanding the pain behind it.” (P1), “She has to give the trophy to me” (P4), “That the teacher will allow the student to get inside the class for the same scenario” (P9), and “And the lightning for a wish that I have the person knows the intensity of it” (P12). These narratives suggest that revenge, in these instances, served to restore emotional balance and validate personal experience through imagined reciprocity. There was strong convergence in the messages elicited by both drawings and narratives from participants whose revenge fantasies fell within this theme.

### Theme 2: Restoring Moral Order Through Indirect or Inevitable Justice

Revenge fantasies were also expressed as a restoration of moral order and justice delivered indirectly, through the passage of time or external forces that would eventually lead perpetrators to recognize their actions. Experiences of betrayal, shame, and emotional conflict stemming from unjust events were evident in both drawings and narratives. Participants were not positioned as active agents within these revenge fantasies; rather, they imagined outcomes in which consequences unfolded without their direct involvement. This appears to be linked to the nature of the injustices, which were often experienced as overwhelming and left participants feeling unable to confront the perpetrator directly. In cases involving severe violations, such as abuse, this theme also manifested as a need for protection and disclosure. Participants imagined telling trusted figures and having the perpetrator removed from their environment, emphasizing safety and validation. Here, revenge was not directed toward harming the perpetrator, but toward restoring what was absent at the time of the event—namely, voice, support, and protection.

**Fig. 3.**
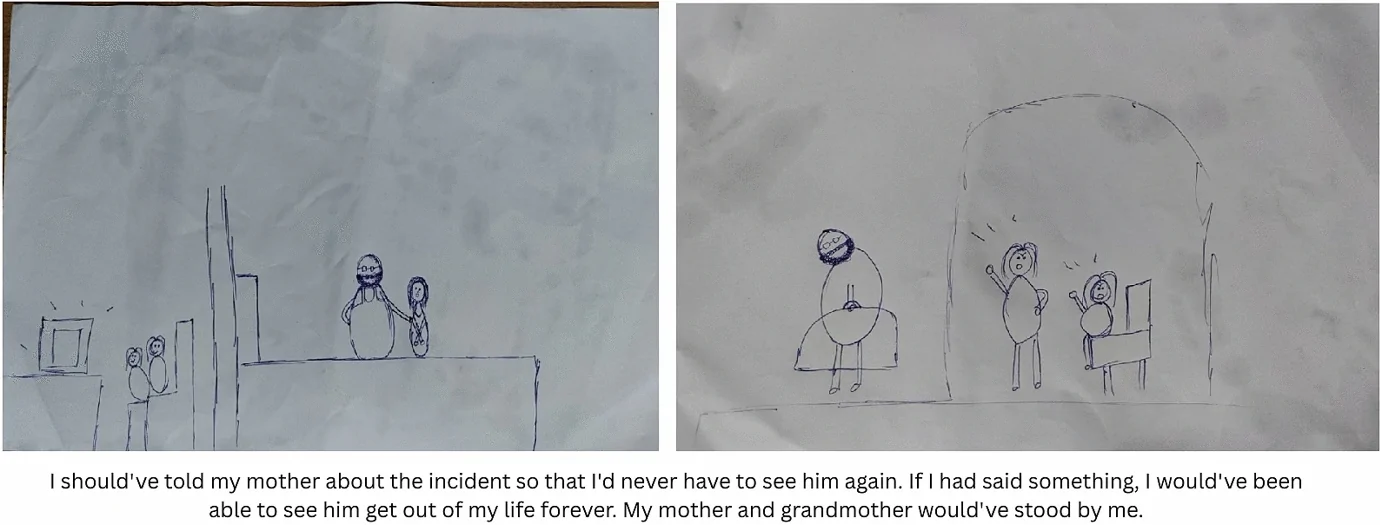
Unjust Event (left) and Revenge Fantasy (right) drawings of participant aged 20 years﻿

**Fig. 4.**
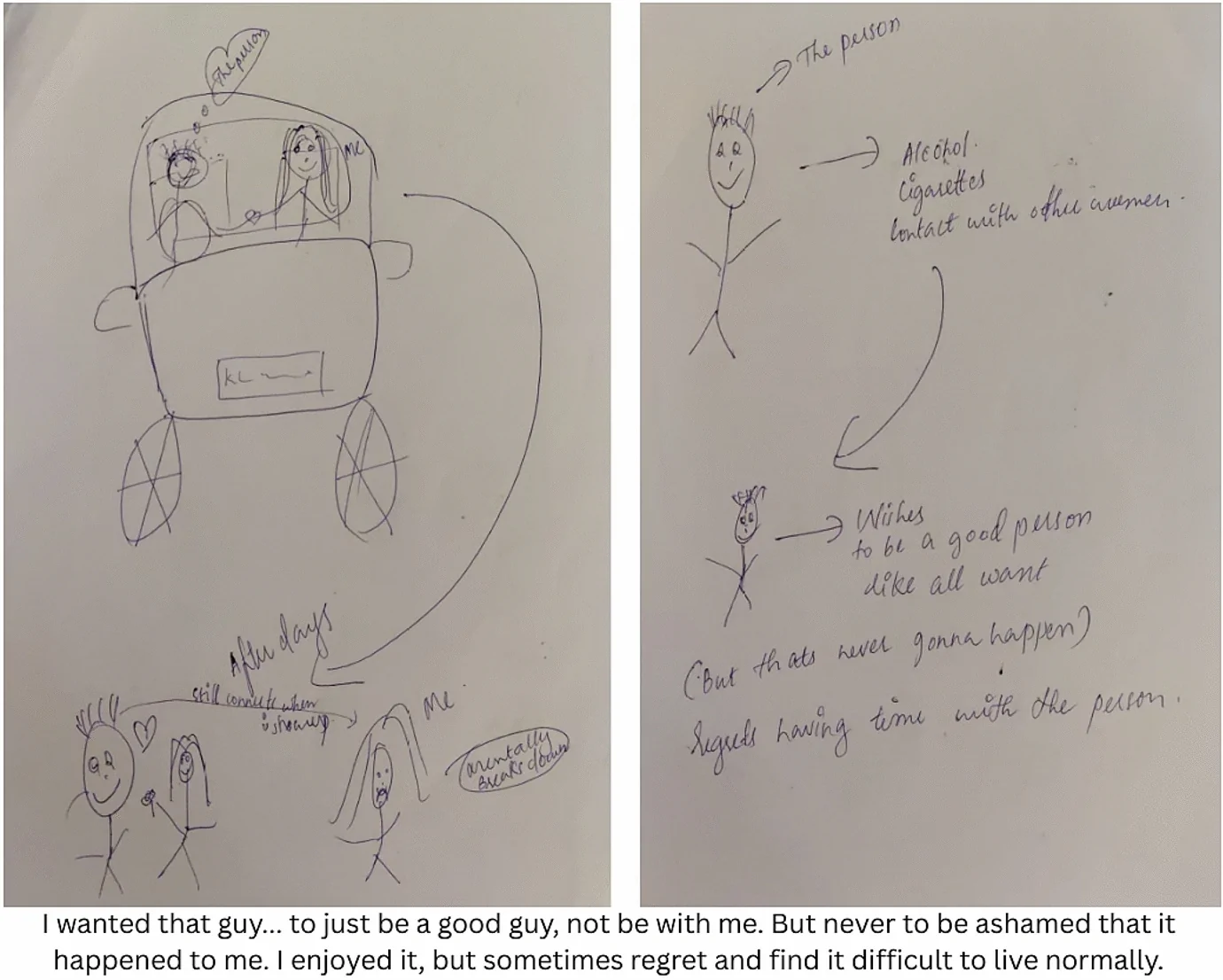
Unjust Event (left) and Revenge Fantasy (right) drawings of participant aged 18 years﻿

A distinct characteristic of the drawings within this theme was their temporal or sequence-based quality, resembling a progression of events (see Figs. 3 and 4). Participants depicted transitions across time, using symbols such as movement through space (e.g., vehicles), domestic settings, or the aging of figures. Visual elements such as boundaries, hierarchical positioning, and inclusion or exclusion corresponded with narratives emphasizing fairness and moral resolution. At the same time, drawings often relied on symbolic representations of power, suggesting that participants experienced injustice not only as interpersonal but also as structurally embedded. Narratives within this theme reflected a fluid and sometimes fragmented emotional process. Participants moved between conflicting positions, as illustrated in statements such as, “I wanted that guy whom I mentioned to just be a good guy, not be with me. But never to be ashamed that it happened to me. I enjoyed it, but sometimes regret and find it difficult to live normally…” (P11) and “But thanks to God, I learned to forgive them and work on myself and things that I love… I am happy now, but will never want any child to go through such a trauma.” (P14). These accounts reflect oscillations between hurt, reflection, and attempts at resolution. Drawings and narratives were only partially convergent in these cases. While both conveyed relational betrayal, emotional conflict, and a desire for moral change in the perpetrator, the narratives revealed a greater degree of ambivalence and contradiction that was less explicitly visible in the drawings. This suggests that drawings may capture a more stable or resolved representation of meaning, whereas narratives reveal ongoing emotional negotiation. Overall, revenge within this theme reflects a process of moral and relational recalibration, in which justice is imagined through restoration of balance, rather than through direct retaliation.

### Theme 3: Reclaiming Power Through Aggression and Achievement

For some participants, revenge fantasies functioned as a means of reclaiming power within disrupted power dynamics, either through imagined elimination of the perpetrator or through personal achievement. In these cases, the focus shifted toward either transforming the self through success or removing the source of harm entirely. In instances involving severe violations, such as sexual abuse, drawings and narratives reflected intense anger, frustration, and a desire for the perpetrator to experience physical harm or irreversible consequences. Participants who experienced chronic invalidation or rejection often expressed a desire for revenge through achievement, success, or personal growth, framing these outcomes as means of countering the original harm. For example, participants described intentions to “prove” others wrong or to succeed despite discouragement, suggesting that revenge was enacted through reclaiming self-worth and agency. In more complex cases, participants demonstrated dual responses, combining outward expressions of anger with inwardly focused coping and self-repair strategies.

**Fig. 5.**
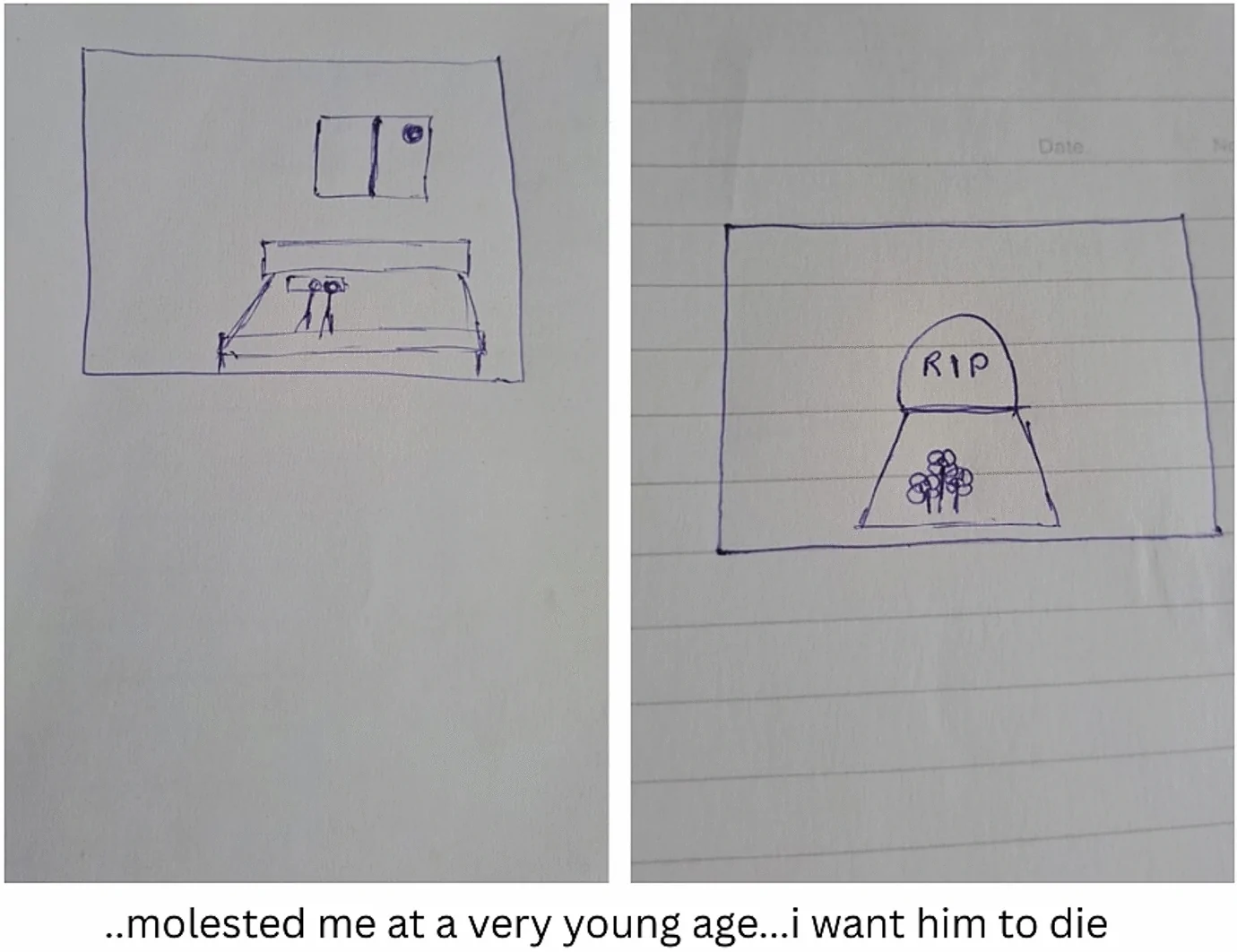
Unjust Event (left) and Revenge Fantasy (right) drawings of participant aged 27 years﻿

**Fig. 6.**
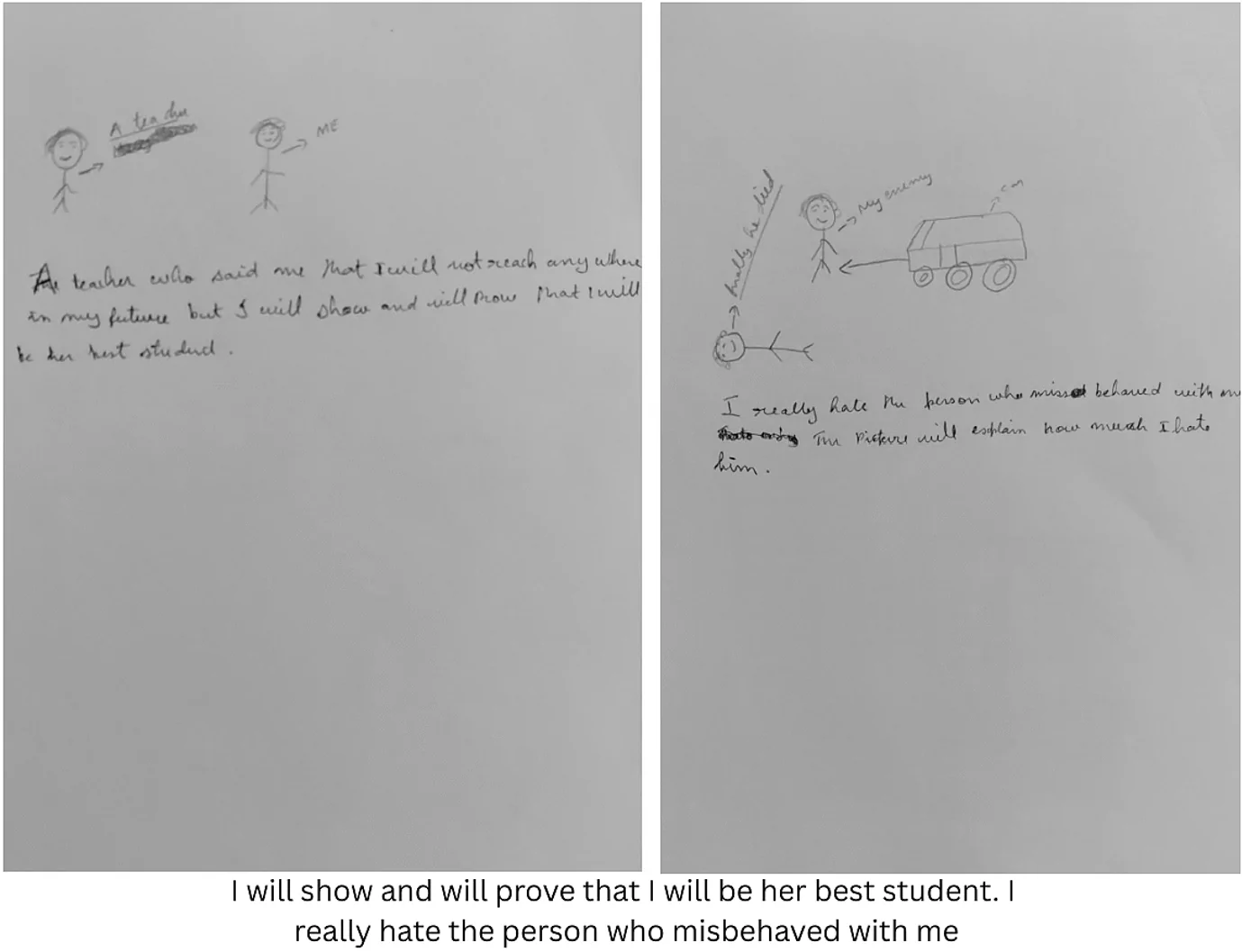
Unjust Event (left) and Revenge Fantasy (right) drawings of participant aged 19 years﻿

Drawings in these cases often depicted direct, action-oriented, or aggressive scenarios, sometimes supported by written labels or thought bubbles that clarified intent. This directness suggests a need for explicit communication of experiences that are otherwise difficult to articulate. Visual elements such as boundaries, lines, and enclosed spaces conveyed restriction, containment, and the impact of gendered or power-laden experiences (see Figs. 5 and 6). Narratives similarly reflected this intensity, often through brief and direct statements such as, “Everyone… causing the abuse [should be] treated by the society with punishment with beating,” and “I really hate the person who misbehaved with me.” Across these cases, there was strong convergence between drawings and narratives in conveying anger and the desire for control or resolution, particularly in instances involving the elimination of the perpetrator. However, partial divergences also emerged. In some cases, drawings depicted explicit acts of aggression, while narratives emphasized achievement, determination, and proving oneself. This suggests a tension between more immediate, unfiltered expressions of anger, and more socially mediated or adaptive responses. Such patterns indicate that participants may simultaneously hold multiple strategies of responding to injustice, reflecting an ongoing negotiation between aggressive impulses and constructive forms of self-repair. Overall, revenge in this theme reflects an effort to reclaim agency, either by directly eliminating the source of harm or by rebuilding the self in response to it.

### Theme 4: Revenge as Withdrawal, Moral Reflection, and Transformation

A subset of participants expressed revenge through withdrawal, distancing, or a transformation in perspective. In these cases, revenge appeared diffused, indirect, or, at times, relinquished altogether. Some participants demonstrated emotional disengagement, reflected in vague, or generalized narratives and minimal or abstract drawings. This suggests difficulty articulating the experience or a tendency to cope by distancing. In such instances, revenge fantasies were unclear or fragmented, indicating that the primary process may involve avoidance or dissociation rather than retaliation. Other participants expressed revenge indirectly or symbolically, such as by imagining the perpetrator’s future life or moral trajectory. Rather than intervening directly, participants positioned themselves as observers of eventual consequences for the perpetrator.

**Fig. 7.**
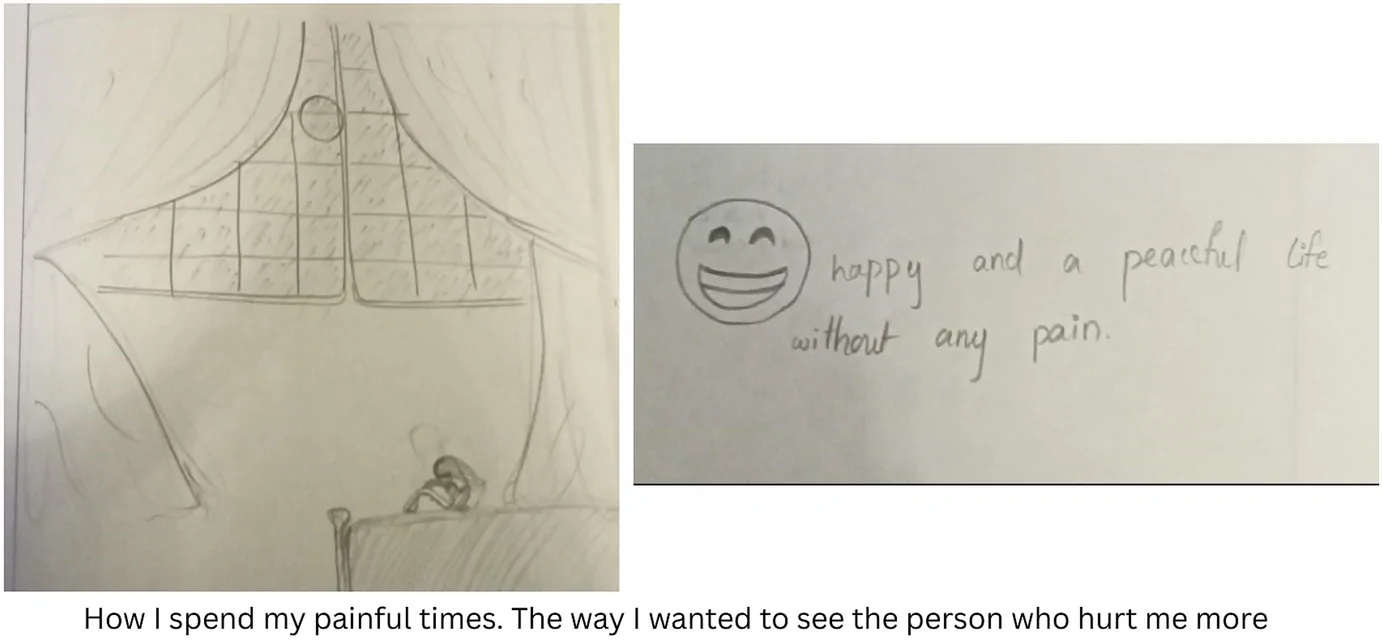
How I spend my painful times. The way I wanted to see the person who hurt me more.

**Fig. 8.**
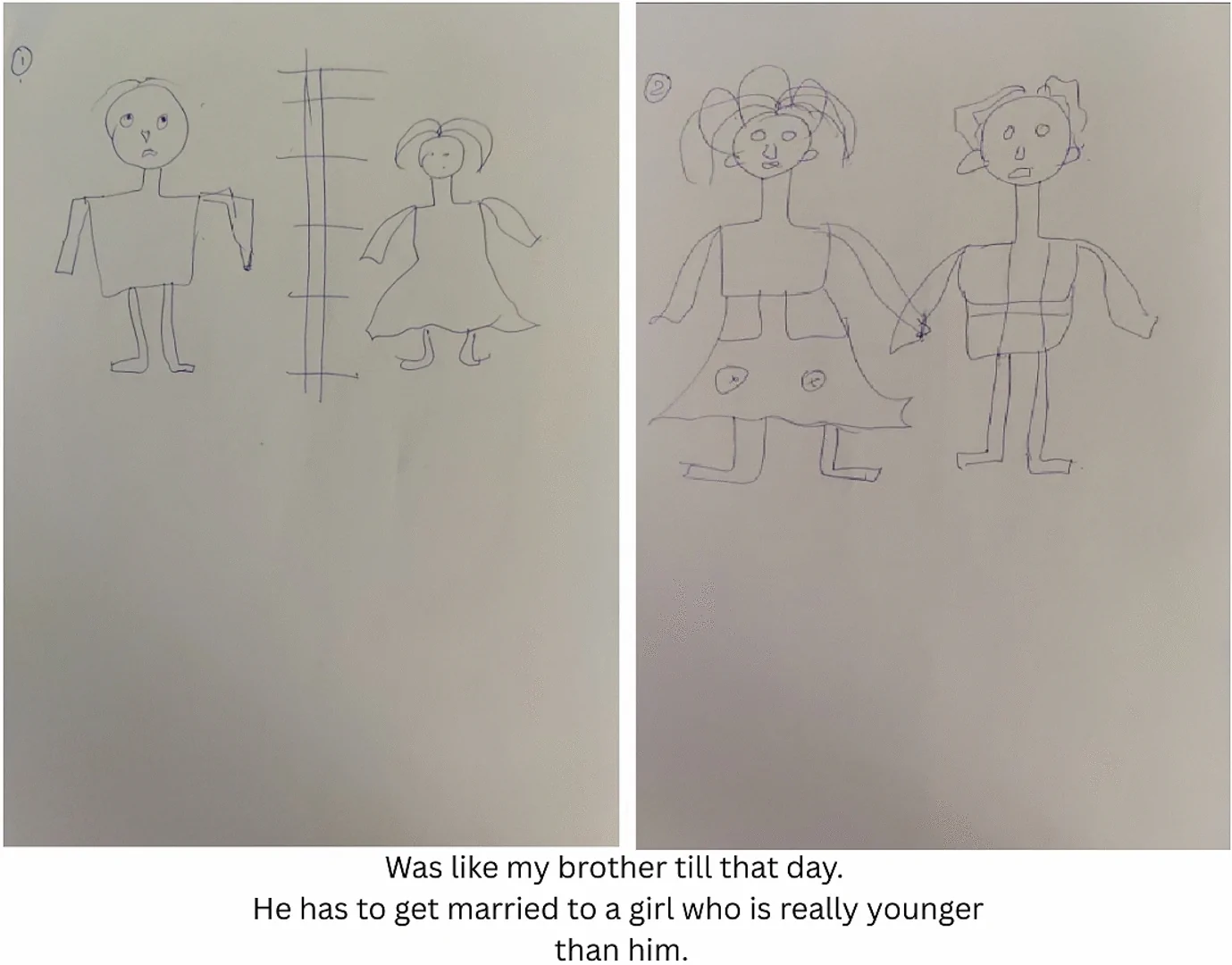
Was like my brother till that day. He has to get married to a girl who is really younger than him.

Drawings within this theme frequently depicted figures that appeared frozen or static, with simplified facial features and minimal contextual detail (see Figs. 7 and 8). These images emphasized isolation, vulnerability, and emotional overwhelm within a dominant or controlling environment. Such representations suggest that the injustices experienced had a lingering psychological impact, with participants appearing “stuck” in the emotional state associated with the event. Correspondingly, narratives often remained vague and non-specific, referring to “painful times” without clearly identifying a source, reinforcing this sense of unresolved or internalized distress. Some of the phrases in the narratives were “How I spend my painful times,” “The way I wanted to see the person who hurt me more,” and “It's not my personal experience, but I'm a person who saw that person who was treated that way.”

However, variation within the theme was evident. In one case, the unjust drawing and narrative were largely convergent, both emphasizing institutional exclusion, lack of transparency, and the participant’s position within an authority structure. In the revenge component, the drawing depicted emotional mirroring alongside passive observation of the perpetrator’s suffering, while the narrative introduced an additional layer of distancing by framing the experience as something witnessed rather than personal. This suggests that the participant managed the impact of injustice by shifting from personal identification to a more detached stance, indicating a coping strategy that combines disengagement with moral evaluation. In another case, the revenge drawing suggested a desire for reconnection and repair through physical closeness, whereas the narrative shifted toward a more indirect and observational stance, focusing on the perpetrator’s future life without explicit action. This discrepancy may reflect an internal tension between a longing for relational repair and an acceptance of distance, suggesting that the participant is navigating an unresolved emotional attachment alongside a need to disengage. Divergences between drawings and narratives were particularly pronounced within this theme. Drawings often preserved raw emotional content or unresolved tension, while narratives reflected more regulated, socially mediated, or at times unclear expressions. This suggests that visual and verbal modalities may capture different stages or layers of processing, with drawings reflecting immediate affective experience and narratives reflecting attempts at cognitive organization or distancing.

## Discussion

The current study adopted a multimodal approach, integrating drawings and narratives, to explore experiences of injustices and associated revenge fantasies among an under-researched population, namely, Muslim women from Kerala, India. The findings highlight that revenge fantasies are not uniform but reflect multiple psychological processes, with a shared thread of participants not positioning themselves as direct agents of revenge. Instead, revenge was imagined, negotiated, or transformed through indirect, symbolic, or internally oriented means.

Revenge fantasies, in the form of reciprocal emotional experience, appeared to function as a means of validating emotional pain and distress arising from unjust experiences. Rather than reflecting a purely aggressive intent, these fantasies can be understood as expressing a need for recognition and acknowledgment, wherein the perpetrator is imagined to experience the same emotional suffering. This suggests an attempt to restore balance within disrupted power dynamics through emotional mirroring. Such processes may be understood in relation to experiences of humiliation and threats to self-worth (Honneth, 2014; Hörnqvist, 2024), particularly in contexts of peer and institutional invalidation. The narratives, as a verbal mode of expression, often appeared direct and concise, leaving little space for repair or negotiation, which may reflect the intensity of emotional experience. This pattern may also be interpreted in light of broader socio-cultural and religious frameworks within which these women are situated, for example, in relation to principles such as Qisas (“an eye for an eye”), which emphasize proportionality in response to harm (Suarning et al., 2025). Within this context, revenge fantasies may be seen as reflecting a need to be seen, heard, and restored in relational terms.

A second pattern that emerged was the conceptualization of revenge as the restoration of moral order through indirect or inevitable justice. According to Quranic teachings, the notion of forgiveness goes hand in hand with justice, especially in contexts of injustices and suffering. Believers are discouraged to tolerate wrongdoing and assured that even if they’re unable to get back at their perpetrator (Qisas), justice will inevitably be done by Allah (Firdous et al., 2024). Quran 45:22 quotes the following: “Allah has created the heavens and the earth with just purpose, and so that everyone is recompensed for what he or she earned, and they will not be wronged.” This appeared apparent in the cases from the current study, where, participants did not imagine themselves as enacting revenge, but rather positioned justice as unfolding through external forces, time, or broader moral systems. This suggests that revenge fantasies may function as a form of psychological repair in contexts where direct confrontation is experienced as difficult or unsafe. Such patterns can be understood in relation to broader belief systems concerning accountability and delayed justice (Belhaj, 2025), as well as psychological frameworks such as the just world hypothesis (Lerner & Miller, 1978), in which individuals maintain the belief that wrongdoings will eventually be corrected. Within the present data, this was reflected in the outsourcing of justice to time, fate, or higher powers. The present sample was relatively homogeneous and highly educated, representing one of the more socio-economically mobile and educationally engaged Muslim populations in South India (Alam, 2022). At the same time, many Muslim girls in Kerala are exposed from a young age to religious and cultural teachings through institutions such as Madrasa education, where values such as *Sabr* (patience), *Haya* (modesty), and restraint are often emphasized. One possible interpretation is that the tendency to imagine justice through indirect, symbolic, or inevitable forces reflects a negotiation between educational empowerment and personal agency on the one hand, and culturally embedded values of patience, restraint, and moral conduct on the other hand. In this context, revenge may be imagined not through direct retaliation, but through proxy or external routes by which the injustice is ultimately addressed. Thus, revenge fantasies may be less about inflicting harm and more about restoring a disrupted moral and social order. Accordingly, the desire for moral restoration appeared closely tied to concerns about social standing, shame, and the need for vindication, suggesting that justice was not only about punishment but also about reclaiming dignity and restoring one's social identity (Sinaga, 2024).

Another key finding relates to the role of revenge fantasies in reclaiming power and control. There appears to be an emerging pattern suggesting that women may experience constraints in expressing anger in the real world, and the processing of the pain associated with injustices happens in the imaginary space (Pasaribu & Ananda, 2022). This was reflected in narratives of participants who brought up sexual abuse in their Trauma Events Checklist as well as in the data (refer Table 1). Both drawings and narratives indicated that participants engaged in imagined scenarios of aggression or personal success to counter experiences of powerlessness. This aligns with power and control frameworks, where individuals seek to restore agency following experiences of subordination (Beebeejaun-Muslum, 2024; Farias et al., 2023). In several cases, particularly those involving sexual abuse, revenge fantasies included elements of direct harm toward the perpetrator, reflecting intense anger and frustration. At the same time, achievement and success emerged as alternative pathways for restoring self-worth, suggesting that participants were negotiating between destructive and constructive modes of response. Within patriarchal contexts, where direct confrontation may not be feasible, such alternative expressions of agency may serve important self-regulatory functions. Revenge fantasies, in this sense, may be understood as attempts to re-establish dignity and self-respect (Goldner et al., 2019), with achievement functioning as a symbolic assertion of power and a means of repositioning oneself within social hierarchies (Jaroenkajornkij et al., 2025; Mccarthy et al., 2006).

The theme of withdrawal, moral reflection, and transformation highlights a more internally oriented response to injustice. Consistent with trauma literature, intense and overwhelming experiences may be associated with emotional shutdown, distancing, dissociative processes, or difficulties articulating experience (Clancy & Egan, 2022; Zukerman et al., 2023). Within art-based trauma research, minimal, fragmented, or altered visual representations have been linked to dissociative and distancing processes. For example, Lev-Wiesel et al. (2024) found associations between dissociation and fragmentation in drawings among survivors of childhood sexual abuse, while Goldner and Frid (2022) reported omissions of body parts, disconnection between figures, and altered figure placement among sexual assault survivors. Similarly, Ram-Vlasov et al. (2019) identified relationships between peri-traumatic dissociation and specific drawing indicators within trauma-focused assessments. Narrative trauma research has likewise shown that traumatic experiences may be communicated through fragmented, emotionally restricted, or temporally disorganized narratives (Kenardy et al., 2007; Van der Kolk & Fisler, 1995). In the present study, several participants produced vague narratives, minimal drawings, and indirect or fragmented expressions of revenge, which may reflect distancing, emotional disengagement, or difficulties articulating the experience. Rather than externalizing anger, participants appeared to engage in internal processing, reframing, or withdrawal. Revenge, in these cases, seemed to transform into reflection, moral evaluation, or disengagement, suggesting a shift from external retaliation toward internal meaning-making. Such responses may also relate to perceived lack of control over outcomes or learned helplessness (Rezaei et al., 2025; Shahir et al., 2025), whereby withdrawal functions as a protective or adaptive response.

A key contribution of the present study lies in the integration of drawings and narratives as complementary modes of expression. The findings suggest that drawings often captured more immediate, affective, and implicit aspects of experience, including raw emotional intensity and unresolved tension, whereas narratives reflected more structured, regulated, and socially mediated accounts. This distinction highlights how different modalities may represent different stages or layers of psychological processing. In cases where drawings and narratives were convergent, participants appeared to have a clearer and more consolidated understanding of their experiences and responses. In contrast, divergences between visual and verbal expressions revealed internal conflicts, ambivalence, or evolving interpretations of injustice and revenge. For instance, aggressive impulses depicted in drawings were sometimes accompanied by narratives emphasizing achievement or restraint, suggesting a tension between unfiltered emotional responses and socially acceptable forms of coping. Similarly, shifts from revenge-oriented imagery to reflective or growth-oriented narratives may indicate a temporal evolution in meaning-making.

Across themes, revenge was rarely imagined as indiscriminate retaliation. Instead, participants frequently imagined acknowledgment, validation, protection, moral correction, social recognition, or restoration of dignity. This suggests that the central concern was often not the perpetrator's suffering itself, but the repair of psychological, relational, or social injuries created by the injustice. Revenge fantasies, therefore, appeared to function as attempts to restore disrupted moral and interpersonal worlds rather than simply enact punishment. A recurring pattern across themes was the outsourcing of justice to external agents, including authority figures, family members, social systems, time, fate, or divine processes. Participants frequently imagined justice being restored without direct personal action, suggesting that revenge was often conceptualized as something that unfolds rather than something personally enacted. Although participants shared a Muslim identity, religious explanations were not consistently foregrounded in either the drawings or the narratives. Instead, experiences of injustice were primarily framed in terms of interpersonal, relational, and emotional concerns.

Overall, the findings suggest that revenge fantasies among Indian Muslim women are multifaceted and dynamic, functioning less as intentions to harm and more as processes of restoring meaning, agency, and emotional balance. The use of drawings alongside narratives offers a nuanced understanding of these processes, revealing both expressed and unarticulated dimensions of experience. Rather than representing a fixed response, revenge emerges as a fluid psychological phenomenon that may shift across contexts, time, and modes of expression. While the present study offers preliminary insights into the potential role of socio-cultural and religious contexts in shaping these processes, further research is needed to more systematically examine these influences, particularly regarding how beliefs about justice, accountability, and gender norms interact with individual experiences of injustice.

## Limitations

Several limitations should be acknowledged. First, the study is based on a relatively small and contextually specific sample of Indian Muslim women, and the findings are, therefore, not intended to be statistically generalizable. The goal was depth rather than breadth, and the analysis should be read as illuminating culturally situated processes of meaning-making rather than making claims about all Muslim women or all experiences of revenge. This is especially important given the diversity within Muslim communities in relation to class, education, region, religiosity, marital status, and exposure to violence. Second, the use of drawings and narratives, while a strength, also introduces interpretive complexity. The interpretation of both, therefore, depends on a careful and reflexive analytic process. What is offered here is not the final truth of participants’ revenge fantasies, but an interpretative account grounded in their representations and narratives. Third, because the study focuses on expressed fantasies and representations, it cannot make claims about the long-term trajectory of these fantasies, including whether they later diminish, intensify, or undergo transformation, or whether they relate to mental health outcomes over time. A longitudinal design might have helped clarify whether revenge fantasies function primarily as temporary affective release, enduring symbolic resistance, or part of a broader coping pattern. The findings must be understood in light of possible constraints on disclosure. Given the moral, relational, and religious sensitivities surrounding anger, revenge, and female expression, some participants may have softened, displaced, or reformulated aspects of their experience in ways that felt more socially or morally acceptable. In this sense, even the drawings and narratives may represent negotiated versions of inner life rather than direct access to it. Yet this too is meaningful, because the negotiation itself reflects the very cultural tensions the study sought to explore. The sample consisted exclusively of university students and, therefore, reflects the experiences of a relatively educated subgroup of Muslim women. Future studies should include women from more diverse educational, occupational, and socio-economic backgrounds to better understand the range of meanings attributed to injustice and revenge across different contexts. Finally, while the study foregrounds culture, religion, and gender as shaping contexts, these dimensions should not be treated as exhaustive explanations. There may be other important influences, including personality, trauma history, family dynamics, political exposure, or previous experiences of injustice, that also shape how revenge is imagined and expressed. Future research could also build on the present work by examining these intersections more explicitly and by comparing how revenge fantasies are negotiated across different groups of women, generations, levels of education, and religious and socio-political contexts.

## Implications

The implications of the study are conceptual, methodological, and practice-oriented. Conceptually, the findings invite the understanding of revenge fantasies as culturally and relationally situated responses to experiences of injustice. Within this sample of Muslim women from Kerala, revenge was frequently imagined through acknowledgment, vindication, moral correction, achievement, and the restoration of dignity rather than through direct retaliation. This suggests that revenge fantasies may serve not only as responses to harm but also as attempts to repair disrupted moral, relational, and personal worlds.

Methodologically, the study supports the value of integrating drawings and narratives when examining emotionally complex and morally ambivalent experiences. The findings demonstrate how visual and verbal modalities can reveal different layers of meaning, particularly in relation to experiences involving shame, anger, betrayal, moral conflict, and social constraint. The convergence and divergence between drawings and narratives provided insights that may not have been accessible through either modality alone.

At the level of practice, the findings highlight the importance of attending to the specific meanings embedded within revenge fantasies rather than assuming that all revenge-oriented thoughts reflect a desire to harm. For the women in this study, fantasies frequently centered on being acknowledged, believed, vindicated, supported, or morally restored. Such findings suggest that practitioners working with women who have experienced injustice may benefit from exploring how revenge fantasies relate to broader concerns surrounding dignity, recognition, agency, and social belonging. In particular, the findings underscore the value of understanding revenge fantasies within the relational and moral contexts in which they arise, rather than viewing them solely through the lens of aggression or risk**.**

The findings may also be relevant to community-based and preventive interventions. If imagined revenge often reflects unmet needs for recognition, justice, and voice, interventions may benefit from creating opportunities for individuals to safely express, narrate, and symbolically process experiences of injustice. Art-based approaches, narrative practices, and culturally responsive psychosocial interventions may be particularly useful in supporting these processes.

## Conclusion

This study sought to understand how Indian Muslim women experience injustice and express revenge fantasies through drawings and narratives within their socio-cultural and religious worlds. Taken together, the findings suggest that revenge fantasies among these Muslim women from Kerala were concerned with repairing the moral, relational, and personal disruptions caused by injustice. Across the drawings and narratives, participants frequently imagined acknowledgment, vindication, moral correction, achievement, or the restoration of dignity, rather than direct retaliation against perpetrators. What emerged was not merely anger, but a more layered struggle involving humiliation, silencing, moral conflict, injured dignity, and the wish to reclaim voice, balance, recognition, or power in the face of injustice.

A central contribution of the study is to show that revenge fantasies may function as a psychologically meaningful internal space in which experiences of injustice can be revisited, negotiated, and reimagined. Notably, revenge was rarely imagined as indiscriminate retaliation. Instead, participants frequently envisioned acknowledgment, moral correction, vindication, social recognition, or the restoration of dignity, suggesting that imagined responses to injustice were negotiated within relational and moral frameworks rather than outside them. For several participants, the desired outcome was not necessarily the perpetrator's suffering but rather being believed, being seen, having one's pain acknowledged, or restoring a sense of self-worth that had been damaged by the experience. In this sense, revenge fantasies appeared to operate as spaces in which emotional injuries could be symbolically addressed when direct resolution was unavailable or difficult. At the same time, these fantasies did not appear outside morality; rather, they were negotiated within it. Rather than reflecting simple desires for punishment, many participants imagined perpetrators becoming better people, justice unfolding through external forces, personal success serving as vindication, or trusted others providing recognition and support. Such findings highlight the importance of understanding revenge not only as an aggressive impulse but also as a relational and moral response to experiences of injustice.

The study also underscores the value of attending to visual and narrative expression together. Each modality appeared to make different layers of experience available. Read together, the two forms offered a richer view of how injustice is lived, remembered, and reimagined. This is particularly relevant when exploring experiences that may be emotionally complex, socially constrained, or difficult to articulate directly. The convergence and divergence between drawings and narratives revealed aspects of revenge, dignity, moral conflict, and agency that may have remained less visible through verbal accounts alone.

The prominence of themes related to acknowledgment, dignity, vindication, family support, and social recognition may reflect the importance of relational and community contexts within participants' lived experiences. Rather than imagining revenge primarily through direct retaliation, many participants imagined responses that restored damaged relationships, social standing, or moral balance. Such patterns are consistent with the literature describing Kerala Muslim women as actively negotiating experiences of injustice within interconnected familial, social, and moral worlds. Overall, the findings suggest that revenge fantasies among Indian Muslim women are multifaceted and dynamic, reflecting not only responses to harm but also ongoing efforts to restore dignity, recognition, and moral balance. In doing so, the study contributes to a more nuanced understanding of revenge fantasies as culturally and relationally embedded experiences, while demonstrating the value of integrating visual and narrative methodologies in the study of emotionally complex phenomena.

## Competing interest

The authors declare no competing interests.

## Ethics Approval

Ethics approval from University of XXX, Approval Number (Blinded for Review)

Informed Consent

Attained from all the participants in the sample

Consent to Publish

Attained from all the participants in the sample

## Data Availability

Data can be made available from the corresponding author upon reasonable request
